# Molecular biological features of cyst wall of adamantinomatous craniopharyngioma

**DOI:** 10.1038/s41598-023-29664-z

**Published:** 2023-02-21

**Authors:** Chuan Zhao, Ye Wang, Hongxing Liu, Xueling Qi, Zhongqing Zhou, Xianlong Wang, Zhixiong Lin

**Affiliations:** 1 Department of Neurosurgery, Sanbo Brain Hospital, Capital Medical University, Beijing, China; 2 Department of Neurosurgery, The Second Affiliated Hospital, Xiamen Medical College, Xiamen, Fujian China; 3 Department of Neurosurgery, Ditan Hospital, Capital Medical University, Beijing, China; 4 Department of Pathology, Sanbo Brain Hospital, Capital Medical University, Beijing, China; 5 Department of Bioinformatics, School of Medical Technology and Engineering, Key Laboratory of Ministry of Education for Gastrointestinal Cancer, Fujian Medical University, Fuzhou, Fujian China; 6 Department of Neuro-Oncology, Sanbo Brain Hospital, Capital Medical University, Beijing, China

**Keywords:** Cancer genetics, Cancer genomics, CNS cancer, Tumour heterogeneity

## Abstract

The molecular biological differences between cyst walls and those in solid bodies are the foundation of the outcomes. In this study, the *CTNNB1* mutations were confirmed by DNAsequencing; *CTNNB1* expression levels were detected by PCR; the differences between solid bodies and cyst walls in proliferative capacity and tumor stem cell niches were assessed by immunohistochemistry; the effect of the residual cyst wall on recurrence was assessed by follow-up. Mutations in the *CTNNB1* in the cyst wall and the solid body were identical in each case. No differences were found in the transcriptional level of *CTNNB1* between the cyst walls and the solid bodies (*P* = 0.7619). The cyst wall showed a pathological structure similar to the solid body. Proliferative capacity of cyst walls was stronger than that of solid body (*P* = 0.0021), and β-catenin nuclear positive cells (cell clusters) in cyst walls were more than that in solid tumor (*P* = 0.0002). The retrospective 45 ACPs showed residual cyst wall was significantly associated with tumor recurrence or regrowth (*P* = 0.0176). Kaplan–Meier analysis showed there was a significant difference in the prognosis between GTR and STR (*P* < 0.0001).The cyst wall of ACP contained more tumor stem cell niches which could lead to the recurrence. According to the above-mentioned, a special attention to the management of the cyst wall should be paid.

## Introduction

Adamantinomatous craniopharyngioma (ACP) is an intracranial tumor in the sellar region that most common in children aged 5–14 years and less common in adults aged 50–74 years, and less than 2.5 new cases out of 1 million population per year worldwide^1–3^. The tumors often present high-density calcified solid tumor bodies surrounded by low-density cystic fluid. Most ACPs consists of both a solid body and one or more cysts and the differences between molecular characteristics between the two is unclear. Despite of deep location of the tumor and the adjacent vital structures such as hypothalamus, optic nerve, internal carotid artery, people are still undeterred by obstacles on the road to effective management of craniopharyngiomas. In the past, gross total resection (GTR) for ACP was seen as the primary treatment albeit with high rates of serious complications such as optic and endocrinological injury, while with the increasing of the understanding of craniopharyngioma, the improved treatment such as the Subtotal resection (STR) + radiotherapy can also have a sound outcome similar as GTR^4,5^.

Clinically, the remain of the tumor cells after surgery is the very factor for recurrence of craniopharyngioma^4,6^. This medical practice found that the recurrence rate of these patients who underwent tumor resection with minor cyst wall residual was much higher than those who underwent STR with solid tumor body remaining. In view of this phenomenon, we speculated that there was more active and distinct tumor phenotype in the cyst wall of ACP than that in the solid tumor body of ACP. In the previous studies, the characteristics of the residual tumor tissue, whether derived from the solid tumors or cyst wall components have not been well described. Therefore, it is unclear which residual tumor tissue is more prone to recurrence.

Based on the tumor stem cell hypothesis, it is believed that tumor biological phenotypes affiliate with tumor stem cells. Furthermore, previous studies have confirmed the presence of a fraction of tumor stem cells in the ACP tissue. The biological behavior of ACP was determined by these cell clusters by a series of regulatory mechanisms. Therefore, this study aimed to explore the molecular pathological features of ACP cyst wall (particularly related tumor stem cell clusters and their distinct molecular markers) and the relationship between the cyst wall residue and postoperative ACP recurrence to provide a theoretical basis for recurrence and to support correct treatment of the cyst wall perioperatively.

## Materials and methods

### Cases and specimen collection

The cyst wall (Group A) and solid body component (Group B) of 11 ACP patients were obtained from the surgical resection of tumor tissue in Sanbo Brain Hospital of Capital Medical University, China. The study protocol was reviewed and approved by the hospital ethics committee, and all patients included in the study provided signed informed consent to participate.

The cyst wall is “a thin tissue covering the cystic part of the tumor”. During the operation, the wrapping tumor sac fluid was viewed, thin and breakable. Radiographically, the cyst wall is a thin layer at the edge of the cystic part of the tumor, often enhanced on MR after contrast. The solid body is defined as the part of the tumor visualized as solid during surgical resection. Four cases of normal brain tissue (Group C) were derived from patients with epilepsy surgery or cortical fistula.

The follow-ups of 45 ACP cases were randomly selected from the hospital database, with the consent of the patient or guardian.

### ACP cyst wall and solid tissue pathology and differences in β-catenin, Ki67 expression in cyst wall and solid body

Hematoxylin and eosin (H&E) staining: After surgery, tumor specimens were fixed with 10% buffered formalin and embedded in paraffin. Paraffin blocks were cut into 4 µm sections and stained with H&E.

Immunohistochemistry (IHC): Ki-67 and β-catenin were studied by IHC performed using the streptavidin-peroxidase SP method. Four-micron sections were deparaffinized in xylene and dehydrated through ethanol gradient. Endogenous peroxidase was inactivated with 3% H_2_O_2_, and samples were washed with phosphate buffered saline (PBS) and antigen retrieval with heat was employed. Specimens were incubated with the primary antibody at 4 °C overnight and then rinsed with PBS. Horseradish peroxidase-labeled secondary antibody was added dropwise, incubated at 37 °C for 30 min, and then washed with PBS. The specimen was placed in diaminobenzidine and rinsed with PBS. After dehydration and drying, the slides were sealed. Antibody Ki67 (ZM-0167, 1: 200, Zhongshan Goldenbridge Biotechnology, Beijing, China), antibody β-catenin (ZM-0442, 1:200, Zhongshan Goldenbridge Biotechnology, Beijing, China). Whole slide images (WSIs) were obtained with scanner (PANNORAMIC MIDI II, 3DHISTECH, Budapest, Hungary), morphometric data were obtained (×40 amplification option) using CaseViewer 2.4 (64-bit version for Windows; 3DHISTECH; Budapest, Hungary). For β-catenin staining, if the cell structure was brown, the part (membrane, plasma or nucleus) was considered positive, the percentage of β-catenin nuclear positive cells counted through 5 random fields of vision. For Ki67 staining, the percentage of positive cells were counted through 3 random fields of vision with obvious Ki67 nucleus staining positive cells, and the largest percentage was taken as the Ki67 labeling index of the sample, and then performed statistical analysis.

To determine the expression relationship between CD44 (1:200, abcam, ab189524, UK) and β-catenin (1:200, Maixin Biotechnology, MAB-0754, China), double immunofluorescence staining was employed on serial tissue microarray sections using goat anti- mouse (1:500, Beyotime, Alexa Fluor^®^ 488, A0423, China) and goat anti-rabbit (1:500, Abcam, Alexa Fluor® 594, ab150080, UK) secondary antibodies. Nuclei were counterstained using 40, 6-diamidino-2-phenylindole (DAPI, Sigma-Aldrich).

### Analysis of exon 3 mutations in *CTNNB1* gene of ACP cyst wall and solid component by whole exon sequencing (WES)

High molecular weight genomic DNA was extracted from tumor tissue following the established protocol of cellular/nuclear lysis and protein precipitation so as to separate DNA in the supernatant which was then precipitated with isopropanol and washed with 70% ethanol and redissolved in rehydration buffer. WES was carried out by Berry Genomics Company (Beijing, China) using Agilent-V6 and BBG-39 M liquid-phase chip capture systems for human exon capture library building. Quality control of the library was carried out with Qubit2.0 for preliminary quantification, and Agilent 2100 for checking insert size of the library. Paired end sequencing was done with the Illumina NovaSeq Sequencing System (Illumina, San Diego, CA, USA).

Sanger sequencing: The target sequence of the next generation sequencing is exon3 of *CTNNB1*, which is located at 41223526–41224753, with forward primer, 5′-GGCTGTCTTTCAGATTTGACTTTAT-3′, and reverse primer 5′-TATTTGCTATCCTAAATGGTAAAAG-3′. Sequencing reaction system: 5 µl total system, sequencing 2 µl Primer (3.2 µM), purified PCR products 1–3 µl, BigDye Mix kit 1 µl. Reaction cycle conditions: 94 °C for 1 min; 28 cycles: 94 °C for 20 s, 50 °C for 10 s, 60 °C for 4 min; and 4 °C for 30 min. Sequencing was performed using an Applied Biosystems 3730 xl sequencer (Applied Biosystems, Beverly, MA, USA). The sequencing results were analyzed by DNA Sequencing analysis software, and interpreted by Sequencing Analysis 5.2.0 software (Applied Biosystems, Beverly, MA, USA), and then compared by Sequencher 5.1 software package (Gene Codes Corporation, Ann Arbor, MI USA, http://www.genecodes.com).

### Analysis of difference in *CTNNB1* expressions of ACP cyst wall and solid component

Total RNA extraction: Trizol total RNA extraction reagent (RNASure Reagent, Cat # 2102) purchased from Genenode Biotech Co., Ltd (Beijing, China) was used for total RNA extraction with the manufacturers’ instructions provided.

Real-time PCR: First Strand cDNA Synthesis Kit from Genenode Biotech Co., Ltd (Cat # 4202, Beijing, China) was used for reverse transcription according to manufacturer’s instructions. *HPRT1* was used as internal control. The primers used for PCR were as follows: for *CTNNB1* forward primer: 5′-CCCACTAATGTCCAGCGTTTG-3′, and reverse primer: 5′-CCACCTGGTCCTCGTCATTTA-3′; for HPRT1 forward primer: 5′-TGAGGATTTGGAAAGGGTGT-3′, and reverse primer: 5′-GAGCACACAGAGGGCTACAA-3′. Amplification was performed using 2 × SYBR Green qPCR Mix (Antibody) (Genenode Biotech Co., Ltd, Cat # 4302). The relative quantitative analysis of the data was performed using a FS384 real-time PCR system type fluorescence quantitative PCR instrument and 2^−△Ct^ method.

### The relationship between recurrence of ACP after surgical treatment and the residual cyst wall

We performed a prognostic analysis of 45 patients with ACP who underwent surgery at our center.Standard total tumor resection was carried out for all patients using an intraoperative microscope and was confirmed by independent unbiased experienced neuroradiologists. The relationship between the residual cyst wall and postoperative recurrence was studied by comparing associations between total resection under the microscope, postoperative MRI image characteristics and postoperative recurrence.

### Statistical analysis

Statistical analysis was performed using GraphPad Prism 8 (GraphPad Software, Inc., San Diego, CA). Comparisons of mRNA levels of *CTNNB1* of 3 groups of tissues, the percentage of β-catenin nuclear positive cells and Ki67 labeling index (Ki67%) were performed by Mann–Whitney test. Kaplan–Meier analysis and Hedge's g between the two groups were performed by R software (version 4.1.1)^7^.Differences with *P* < 0.05 were considered statistically significant.

### Ethics approval and consent to participate

All procedures performed in studies involving human participants were in accordance with the ethical standards of the institutional and/or national research committee and with the 1964 Helsinki Declaration and its later amendments or comparable ethical standards. The study was reviewed and approved by the human subjects’ institutional review boards of Sanbo Brain Hospital of Capital Medical University (SBNK-YJ-2020-014-01). Written informed consent was obtained from the families of all patients whose tumor and/or blood samples were collected.

## Results

### Patients’ demographics, clinical characteristics and case follow-up data

Patients’ demographics, clinical characteristics and case follow-up data are shown in Table 1.

**Table 1:** Summary of clinical data.

Case	Sex	Age(years)	Pathologic diagnosis	Surgical Approach	Resection extent	Outcome	Recurrence	WES	Sanger sequencing	*CTNNB1* level
1	Female	16	ACP	Transcallosum approach	GTR	Alive	No	Yes	Nd	No
2	Female	6.83	ACP	Pterional approach	GTR	Alive	No	Yes	Nd	No
3	Male	14	ACP	Frontobasal interhemispheric approach	GTR	Alive	No	Yes	Nd	No
4	Female	3.08	ACP	Frontobasal interhemispheric approach	GTR	Alive	No	Yes	Nd	No
5	Female	4.92	ACP	Transsylvian approach	GTR	Alive with disease	No	Yes	c.110C > T, p.S37F	Cyst wall and solid tumor
6	Male	34	ACP	Frontobasal interhemispheric approach	GTR	Alive	No	Yes	c.132_170del	Cyst wall and solid tumor
7	Male	31	ACP	Frontobasal interhemispheric approach	GTR	Alive	No	Yes	c.94G > A, p.D32N	Cyst wall and solid tumor
8	Male	65	ACP	Frontobasal interhemispheric approach	STR	Alive	No imaging review	Yes	c.98C > G, p.S33C	Cyst wall and solid tumor
9	Male	46	ACP	Frontobasal interhemispheric approach	GTR	Alive	No	Yes	c.109 T > G, p.S37A*	Cyst wall
10	Male	21	ACP	Pterional approach	GTR	Alive	No	Yes	No mutation found	Cyst wall
11	Male	8.92	ACP	Frontobasal interhemispheric approach	GTR	Alive	No	Yes	Nd	Nd

*ACP* Adamantinomatous craniopharyngioma, *WES* Whole exome sequencing, *GTR* Gross total resection, *STR* Subtotal resection, *Nd* no data.

*mutation was only found in cyst wall.

### Surgical procedure

Under the microscope, transcranial surgeries were performed.We first separated the arachnoid membrane outside the cyst wall, and then punctured the thin layer of the cyst wall to release the cyst fluid. After tumor decompression, the thin wall part of the cyst wall and the solid part of the tumor tissue under the cyst wall were taken as research materials under a microscope. Then, the cyst wall was sharply separated from normal brain tissue and other attached structures, pulled then cut piece by piece until it was completely removed together with the solid part of the tumor, and finally complete tumor resection was achieved.No additional optic nerve or hypothalamus damage was caused.

### Imaging characteristics of ACP cyst wall and intraoperative material collection

**Figure 1:**
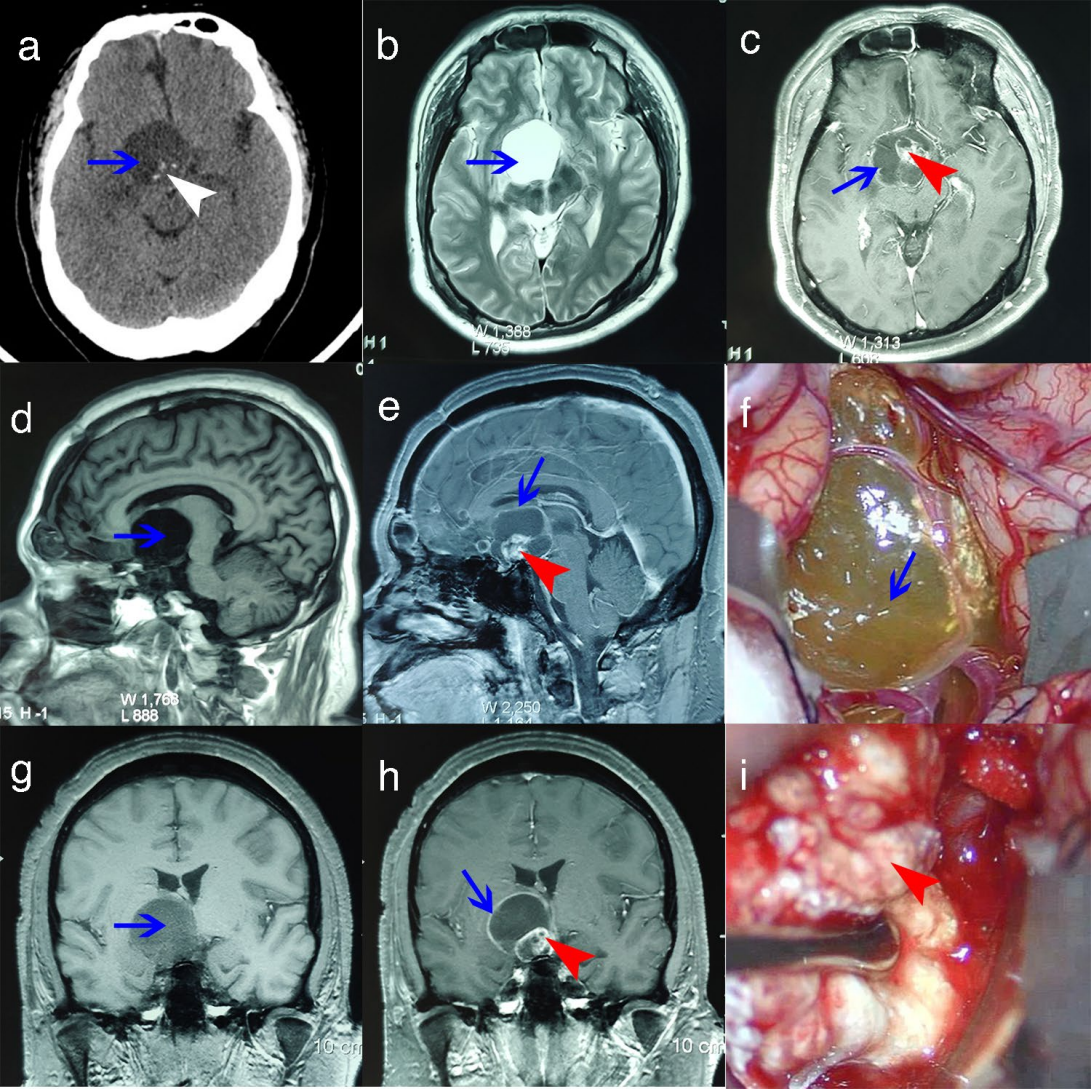
Preoperative and intraoperative imaging of cyst wall and solid body. (**a**) CT scan shows low-density cystic lesion and high-density calcifications (blue arrow, cyst; white arrowhead, calcifications). (**b**) T2-weighted axial MRI shows hyperintense signal lesion (blue arrow: cyst). (**c,e,h**) T1-weighted enhanced MRI shows the peripheral cyst wall and heterogeneous solid body (blue arrow, cyst wall; red arrowhead, solid body). (**d,g**) T1-weighted MRI shows hypointense signal lesions (blue arrow: cyst). (**f**,**i**) Cyst wall and solid body during operation (blue arrow, cyst wall; red arrowhead, solid part).

CT scans (from case 6) of ACP showed a low-density cystic component and a high-density calcified solid component (Fig. 1a). Under MRI (from case 6), the cystic component of the T1 sequence showed a low signal (Fig. 1d, g), and the cystic component of the T2 sequence showed a high signal (Fig. 1b). Under enhanced scanning, the cyst wall and the solid component produced obvious enhanced signals (Fig. 1c, e, h). During the operation, the cyst wall was seen as a translucent film with variable thickness and different degrees of adhesion to and invasion into the surrounding tissue (Fig. 1f). The solid tumor component was solid, tough, and mixed with hard calcifications (Fig. 1i). ACP imaging characteristics revealed that the cyst wall and the solid tumor component had a certain connection or continuity, and the cyst wall was likely to be tumor tissue that maintained continuity with the solid tumor component. During surgical resection, the cyst wall presented more difficulties than the solid tumor component.

### Histopathological characteristics of ACP cyst wall

**Figure 2:**
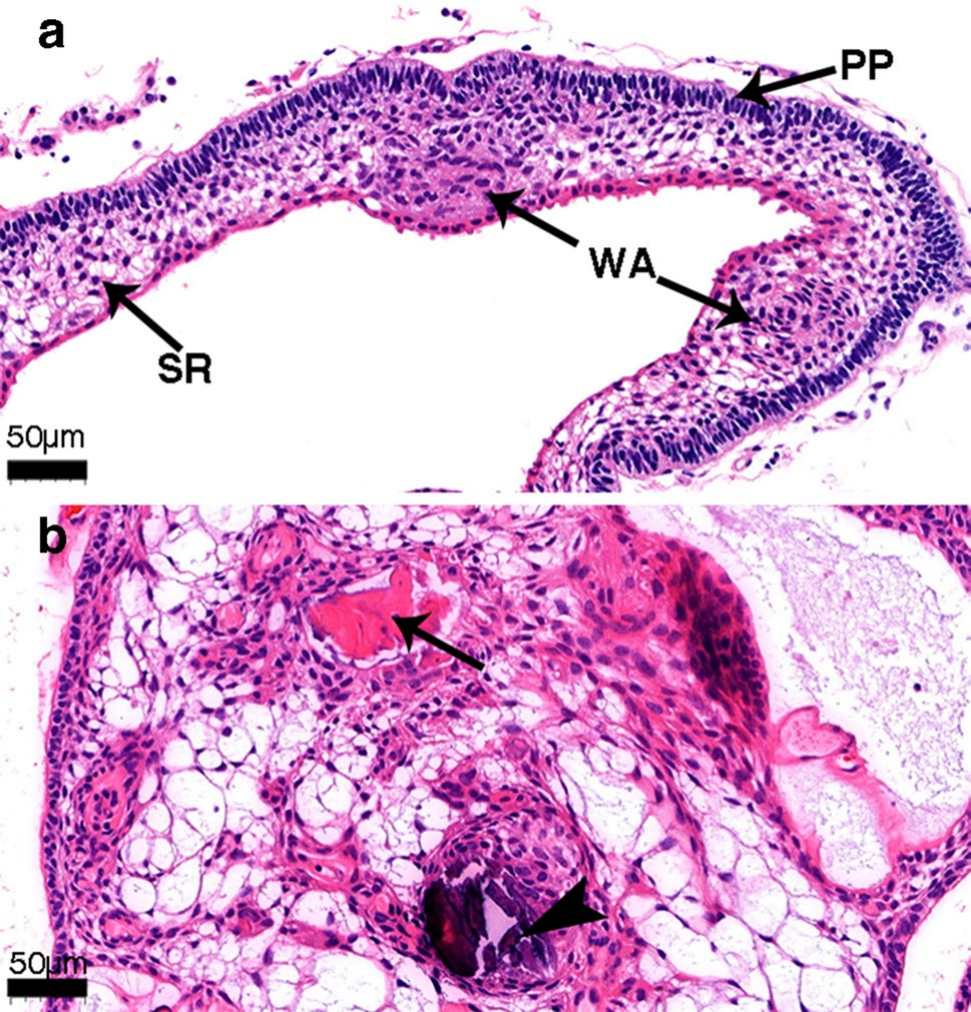
Histopathological features of cyst wall. (**a**) Hematoxylin and eosin (H&E) staining of the cyst wall, showing obvious adamantinomatous craniopharyngioma structure, peripheral palisades, stellate reticulum structure on the inside, and whorl-like array cells. (**b**) Hematoxylin and eosin (H&E) staining of the solid component, with wet keratin (arrow) and calcification (arrowhead) can be seen. (PP: peripheral palisades; SR: stellate reticulum; WA: whorl-like array cells).

H&E staining of all 11 tumor tissues showed that the cyst wall (Fig. 2a) of ACP had similar distribution of tumor cells as those of the solid tumor component (Fig. 2b). For example, the peripheral palisading cells had the characteristics of tumor cells such as nuclear division (Fig. 2a, PP), stellate reticulum (Fig. 2a, SR), whorl-like array cells (Fig. 2a, WA).

### Mutations of *CTNNB1* gene in the cyst wall of ACP

**Figure 3:**
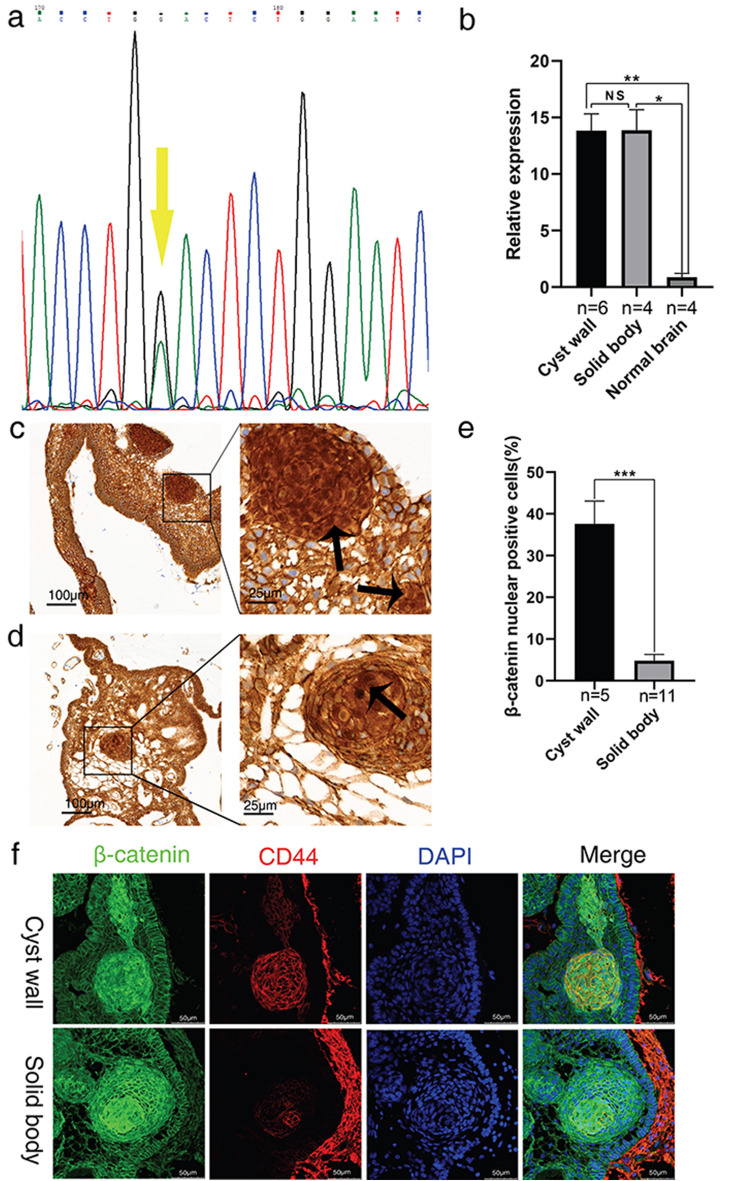
Molecular biological characteristics of the cyst wall. (**a**) Analysis of *CTNNB1* gene in cyst wall tissue by Sanger sequencing reveal missense mutation (yellow arrow). (**b**) There is no significant difference between the cyst wall and solid body. The expressions of *CTNNB1* in the cyst wall and the solid are higher than those in normal brain tissues. The relative mRNA level of *CTNNB1* was calculated by 2^−Δct^ method, *HPRT1* is reference gene. (**c**) The tumor cells of cyst wall are all positive for β-catenin membrane staining, and β-catenin cytoplasm and nuclear staining cells (stem cells) are obvious (arrow). (Immunohistochemistry for β-catenin ,under low-power magnification, scale bar = 100 μm; under high-power magnification , scale bar = 25 μm). (**d**) The tumor cells of solid body are all positive for β-catenin membrane staining, and β-catenin cytoplasm and nuclear staining cells (stem cells) can be seen (arrow) .(Immunohistochemistry for β-catenin ,under low-power magnification, scale bar = 100 μm; under high-power magnification , scale bar = 25 μm). (**e**) Percentage of β-catenin nuclear positive cells is high(median,34.60%) in cyst wall than that in solid tumor(median,4.20%).The results are shown as median with upper quartile;*,*P* < 0.05; **,*P* < 0.01; ***,*P* < 0.001. (**f**) Stem cell niche of cyst wall and solid body. β-catenin showed tumor epithelial structure, CD44 confirmed that the niche of tumor stem cells were β-catenin nuclear positive cells, which were more obvious in the cyst wall than solid body. (Immunofluorescence for β-catenin and CD44, scale bar = 50 μm).

The 11 cases of matched cyst wall and solid tumor component samples provided enough DNA from the cyst wall of 6 cases to perform Sanger sequencing for the matched samples (Table 1). 5 of the 6 cases of cyst wall samples had mutations in exon 3 of the *CTNNB1* gene (Fig. 3a), of which 4 had identical mutations in the solid tumor component. In case 10, however, neither the cyst wall nor the matched solid tumor component had *CTNNB1* mutation (Table 1).

### *CTNNB1* transcription in ACP cyst wall and β-catenin expression

The level of *CTNNB1* transcription was quantified in 6 cases of cyst wall with a sufficient amount of RNA and 4 cases of solid tumor component in comparison with 4 cases of normal brain tissue. The transcription level of *CTNNB1* in the cyst wall was not different from that of the solid body (*P* = 0.7619, Hedge's g = − 0.0116), but was much higher than that of normal brain tissue (*P* = 0.0095, Hedge's g = 4.0210), and similar to that of the solid body when compared to normal brain tissue (*P* = 0.0286, Hedge's g = 4.3252) (Fig. 3b).

The pattern of β-catenin protein expression in the cyst wall of ACP was basically the same as that of the solid tumor component as shown by IHC (Fig. 3c, d). Cells that were stained positive with β-catenin in the nuclei and cytoplasm were mainly cells in whorl-like clusters, while peripheral palisading cells containing β-catenin wrre mainly localized at the cell membrane (Fig. 3c, d). Percentage of β-catenin nuclear positive cells was higher (median,34.60%) in cyst wall than that in solid body (median,4.20% (*P* = 0.0002) (Fig. 3e).

### Stem cell niche of cyst wall and solid body

β-catenin demonstrated tumor epithelial structure, CD44 confirmed that the niche of tumor stem cells were β-catenin nuclear positive cells, which were more obvious in the cyst wall than solid body (Fig. 3f).

### Expression of Ki67 and β-catenin in ACP cyst wall

**Figure 4:**
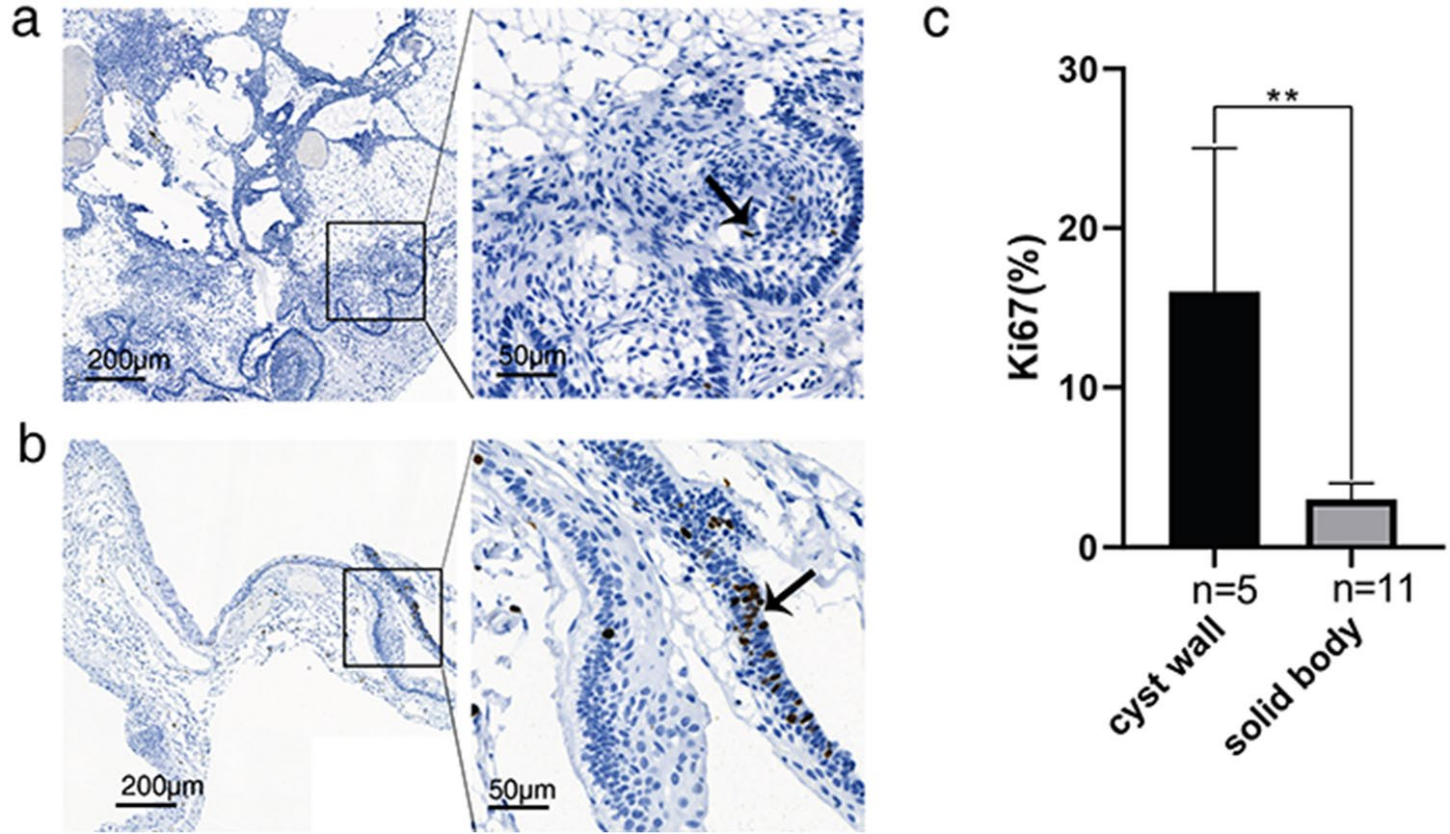
Comparison of Ki67 labeling index of cyst wall and solid body. (**a**) Ki67 stained cells are less in solid body (arrow). (Immunohistochemistry for Ki67, under low-power magnification, scale bar = 200 μm; under high-power magnification, scale bar = 50 μm). (**b**) Ki67 stained cells are mainly distributed in the surrounding peripheral palisades of cyst wall (arrow). (Immunohistochemistry for Ki67, under low-power magnification, scale bar = 200 μm; under high-power magnification, scale bar = 50 μm). (**c**) The Ki67 labeling index of the cyst wall is significantly higher than that of the solid body. The results are shown as median with upper quartile; **, *P* < 0.01.

IHC showed that Ki67 showed positive staining of some nuclei in the peripheral palisading cells of the cyst wall, similar to those of the solid body (Fig. 4a,b). In solid body, the Ki67 labeling index in peripheral palisading cells (median,3%) was lower than that of the matched cyst wall cells (median,16%) (*P* = 0.0021) (Fig. 4c). This suggests that tumor cell proliferation in the cyst wall is more active than that in the solid tumor component.

### Relationship between residual cyst wall and postoperative recurrence

**Figure 5:**
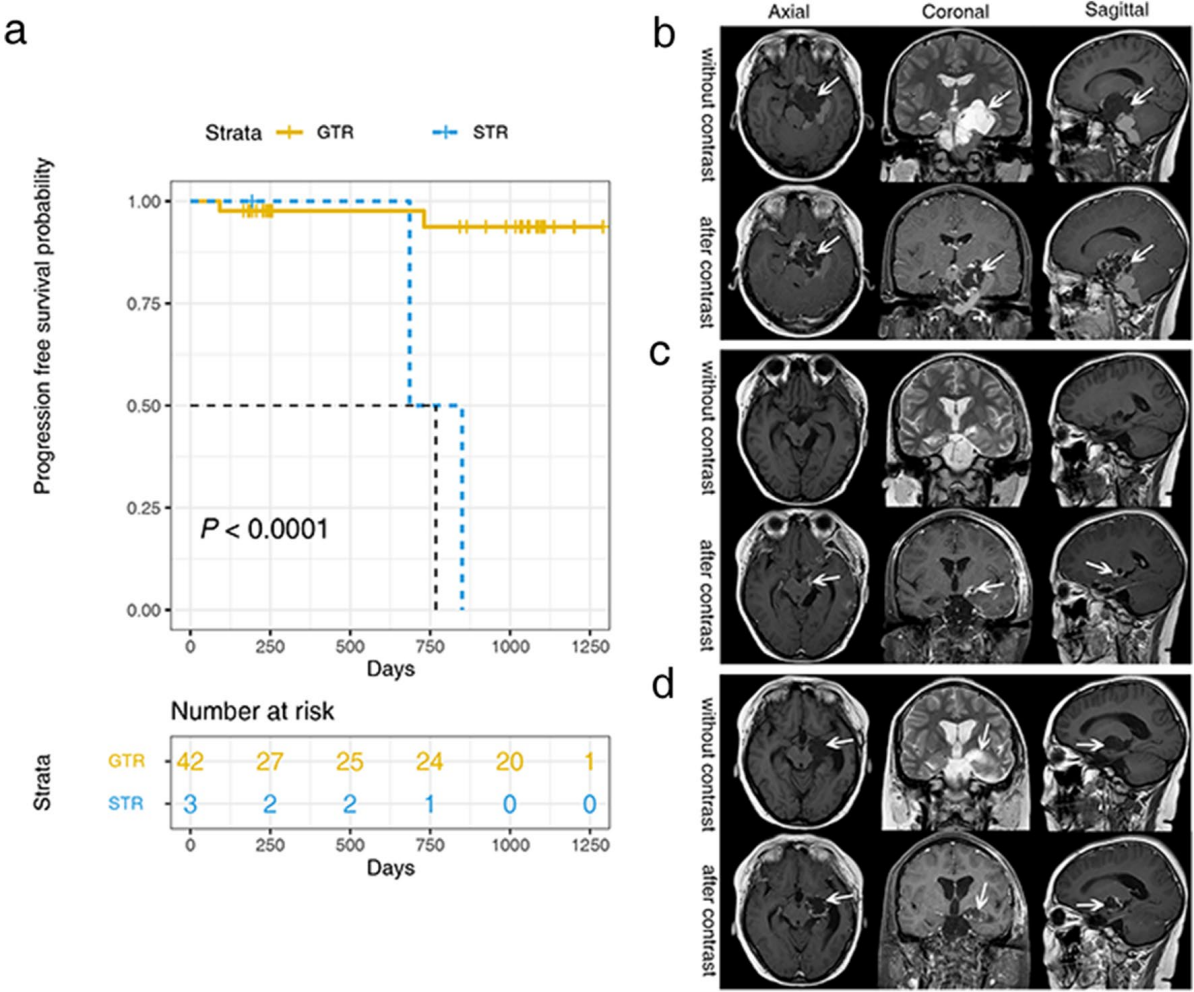
Residual cyst wall led to tumor progression. (**a**) Kaplan–Meier analysis showed significant difference in prognosis between GTR and STR (*P* < 0.0001). (**b**) Preoperative MRI images (arrow: tumor). (**c**) Postoperative MRI images (arrow: residual cyst wall). (**d**) MRI images of tumor recurrence (arrow: tumor regrowth).

A retrospective analysis of the relationship between postoperative recurrence and residual cyst wall in 45 patients with ACP who underwent surgical treatment in Sanbo Brain Hospital was carried out, with 3–43 months follow-up (mean, 23.58 ± 14.25 months). Among these, 42 cases had GTR (resection under the microscope with no residual tumor and confirmed by the first postoperative plain scan and enhanced MRI) including two cases of relapse. The remaining three cases with subtotal resection (STR, a small part of tumor tissue was not totally resected under the microscope, or total resection was not performed under the microscope, but the first postoperative plain scan or enhanced MRI confirmed the presence of residual tumor) included two cases showing progression of the residual tumor and one case with stable disease. The statistical results showed that GTR and STR were associated with tumor recurrence or regrowth (*P* = 0.0176) (Table 2). The analysis made by Kaplan–Meier showed that there was a significant difference in the prognosis between GTR and STR (*P* < 0.0001), and GTR significantly prolonged the time of tumor progression (Fig. 5a).

**Table 2:** Follow-up result of 45 cases.

Resection extent	Outcome	Case number(percent)	*P* value
Gross total resection	Recurrence	2 (4.76%)	
	No recurrence	40 (95.24%)	0.0176*
Subtotal resection	Regrowth	2 (66.67%)	
	No regrowth	1 (33.33%)	

*Fisher's exact test.

Of the two cases that relapsed after GTR, one was detected at the optic chiasm by imaging two years after surgery. By reviewing the intraoperative records at that time, recurrence was suspected to have been caused by the residual cyst wall below the optic chiasm. The other relapsed patient was found to have ectopic recurrence in the frontal lobe due to planting metastasis two years after surgery.

Among the three cases of subtotal resection, two cases were found to have regrowth after surgery. The imaging of the preoperative tumor is shown in Fig. 5b. Intraoperative examination under the microscope showed residual cyst wall, which was confirmed by postoperative imaging (Fig. 5c). The residual cyst wall subsequently increased in size after four months (Fig. 5d). In the following second surgery, no evidence of residual disease was found initially, but recurrence was subsequently found two years later. A small amount of residual cyst wall was seen in the second case under the microscope intraoperatively, and a small amount of residual cyst wall was confirmed by postoperative imaging. Recurrence was found in the follow-up after two years. After the second surgery, no recurrence was found in the ensuing seven years. The third case showed a suspected residual cyst wall under the microscope at the end of surgery but no obvious residual tumor was found by postoperative MRI, and no obvious recurrence was found by surveillance imaging after three months following surgery.

## Discussion

The main histopathological features of ACP are the formation of the surrounding basal cell layer (peripheral palisade of tumor cells); diffuse epithelial stellate cells; enucleated cells with eosinophilic cytoplasm, namely wet keratin; extensive degenerative changes, namely hemosiderin deposits; cholesterol clefts, multinucleated macrophages, and inflammation and calcification, as well as characteristic cell clusters^3,8–10^. In this study, the cyst wall of ACP was found to have typical cell clusters, peripheral palisading tumor cells, stellate reticular cells and other histopathological features similar to those of the solid tumor component. According to tumor biology of craniopharyngioma, it is known that the cyst wall is not a "compressed" solid tissue, but a classic structure produced by the formation of cysts during tumor initiation and progression^3^. Cell cluster is a recognized marker to distinguish ACP from other tumors in the sellar region and squamous papillary craniopharyngioma. It is mainly located at the bottom of the finger-like protuberances which usually invade the tumor epithelium around the tissue^11,12^, and both the cyst wall and the solid tissue follow this distribution. During the surgery, cyst wall is often found to be connected to the solid body and maintains a certain continuity, which further explains the similarity of the histopathological features between the cyst wall and the solid body.

Activation of the Wnt/β-catenin pathway and its downstream pathways are the molecular characteristics of ACP^13^. β-catenin, encoded by the *CTNNB1* gene, is the effector of the canonical Wnt pathway, which controls embryonic development, including axial disposition, organ formation, cell fate, and stem cell self-renewal. Most *CTNNB1* mutations are located in exon 3, which may affect the functional domains of β-catenin, leading to the escape of normal proteasomal degradation (under normal conditions, cytoplasmic β-catenin can be phosphorylated by a degradation complex consisting of adenomatous polyposis coli tumor suppressor protein (APC), glycogen synthetase kinase 3-β (GSK3-β), Axin and casein kinase 1(CK1) and then degraded via ubiquitination). As a result, β-catenin accumulates in the cytoplasm and eventually transfers to the nucleus. In the nucleus, β-catenin possesses the function of a co-transcription factor by interacting with the T-cell factor (TCF) family members to induce downstream abnormal hyperactivation^13–16^. In the present study, sequencing showed that the mutations of *CTNNB1* in the cyst wall were identical to those in the solid body, further indicating that the cyst wall contains tumor cells that are transformed through the same mechanism, and so it was a component of the tumor. There was no mutation in case 10 by Sanger sequencing and whole exon group sequencing, which may be due to technical factors, which is consistent with the previous literature^17^.

The results of PCR suggested that the transcription of *CTNNB1* gene in the cyst wall is higher than that in normal brain tissue and is not significantly different from that of the rest of the tumor. This further suggests that the increase of *CTNNB1* transcription is also an important mechanism of tumorigenesis involving cells in both the cyst wall and the solid tumor.

The histopathological hallmark of human ACP is the accumulation of β-catenin in the nucleus and cytoplasm. These cells can be located as a single cell in the outer palisade cells, but more often gather to form a spiral structure (“cell cluster”)^18–22^. A transgenic mouse model constructed by Martinez et al.^23^ showed that the tumor cells acted as a signal "hub" in ACP, causing downstream changes through paracrine and other mechanisms^23–26^. In the mouse model, β-catenin accumulated in the nucleus and cytoplasm in the nest cell mass and outer palisade cells in both the cyst wall and the solid component with the accumulation was more obvious in the nest cell mass. This indicates that the mechanism of tumorigenesis via activation of Wnt/β-catenin pathway also operates in the cells of the cyst wall. In addition, relevant studies have shown that cases without *CTNNB1* mutation can also have nuclear accumulation of β-catenin in the nest cell mass^20^. Our study are consistent with these previous observations.

Ki67 is a characteristic protein whose positive nuclear staining is associated with proliferation. Ki67 is widely expressed and distributed in craniopharyngioma tumor cells^27^, and its clinical application is controversial^28,29^. Some studies have proposed that Ki67 can be used to predict the progression of craniopharyngioma, so it can provide a reasonable basis for further treatment decisions after surgery^30^. Other studies have failed to show any correlation between Ki67 and tumor recurrence^31,32^. In this study, the proportions of tumor cells and Ki67-positive cells in the cyst wall were higher than those in the solid body, which indicates that the cyst wall may have higher tumor cell activity and residual tissue of the cyst wall should be treated more aggressively after surgery.

Previous studies have suggested that stem cell niches exist in ACP, which are usually located inside the palisade-like epithelium and play an important role in tumor invasion^33,34^. Our study confirmed that this structure exists in the cyst wall and has significant characteristics in the very thin cyst wall, which was verified by immunofluorescence, as shown in Fig. 3. Our study used CD44 as a marker of stem cells and confirmed the presence of stem cells in ACP,which is consistent with previous reports^33,34^. We have shown for the first time that stem cells exist in the cyst wall, which appears to be denser than the solid body. We speculate that the characteristics of stem cells may be related to the biological behavior of the cyst wall. First, stem cells, as the "seeds" of tumors, do not need a large number to support their function. The cyst wall is thinner and the number of cells is less than that of the solid body, but the cells are more refined and the proportion of stem cells is higher. Secondly, the tumor stem cells are related to the invasiveness of the cyst wall. The cyst wall itself is the outermost layer of the tumor, the front line for the tumor to invade the normal structure. This is consistent with the theory that stem cells promote tumor recurrence and metastasis^11,35^. Third, stem cells may indicate the formation of special pathological structures of ACP. Under appropriate conditions, stem cells may be transformed into other layers of tumor cells, such as palisade cells, reticular cells and so on. This may explain that clinically recurrent tumors caused by residual cyst walls may also have solid components. Fourth, it provides basic information for treatment. Surgical treatment should consider removing stem cells, regardless of tumor volume. From the perspective of stem cells, the effect of surgery on recurrence may come from the number of remaining stem cells. Despite the small size of the cyst wall, it cannot be ignored. The resistance of stem cells should be considered in radiotherapy and drug therapy, and the appropriate dose and treatment frequency should be selected^33,36,37^. The search for special targets of ACP stem cells can bring hope for targeted therapy. Immunotherapy may begin with the effect of stem cells on immune cells^38,39^. Despite the high proportion of stem cells in the cyst wall, the space inside the cyst makes the stem cells more easily exposed, which brings hope for comprehensive treatment^40^. In conclusion, the discovery of stem cells in the cyst wall could have a positive impact on treatment.

The latest study has confirmed that β-catenin nuclear stability activates the wnt-signaling pathway and promotes cell proliferation^41^. Our study also confirmed that the cyst wall has more β-catenin nuclear positive cells than the solid body, indicating that the cyst wall has a stronger proliferative capacity.

Previous studies have shown that changes in the tumor microenvironment may indicate a poor prognosis after recurrence of craniopharyngioma^39^. Retrospective analysis showed that the extent of surgical resection of the tumor would affect its prognosis^42^. Partial resection or presence of residual tissue of craniopharyngioma is the main factor affecting tumor recurrence, and total resection has a better prognosis^43–45^. In this study, the average follow-up time was 23.58 ± 14.25 months. Given the invasiveness of craniopharyngioma, the follow-up time was enough to observe the postoperative progress of craniopharyngioma. The clinical follow-up of 45 cases showed that 42 cases with complete tumor resection did not recur, and 3 cases with residual cyst wall recurred. Three cases of recurrence did not recur after total resection. Even if intraoperative assessment of GTR is achieved, postoperative imaging could still indicate the presence of residual cyst wall, which will eventually lead to recurrence in a short time. If part of the cyst wall is left during the operation, it will lead to recurrence. Therefore, great attention must be paid to the management of the cyst wall, which plays an important role in reducing recurrence and improving the prognosis of patients. In short, because the cyst wall has clusters of nuclear-positive β-catenin cells, the residual cyst wall needs to be limited. Thus, treatments like radiation therapy will be needed.

Based on previous experience, radiotherapy for cystic craniopharyngioma has limitations^46–48^. The biological characteristics of the cyst wall should also be considered in radiotherapy of residual tumors after partial resection. Our study shows that the cyst wall contains cell clusters, which are considered to be stem cell clusters with radioresistance characteristics, which may be the reason for the poor effect of radiotherapy^33^. The cyst wall contains denser stem cell clusters (stem cell niches), which lead to radiotherapy failure due to the inherent characteristics of radiotherapy resistance in stem cells. Therefore, if there is residual cyst wall in postoperative imaging (in CP surgery, the solid body is easy to remove, while the cyst wall is thin and easy to be left behind), considering that radiotherapy is limited to stem cells, re-operation should be performed to completely remove the cyst wall. In addition, studies have shown that immune regulation can reduce cystic fluid, which seems to be effective for cystic tumors, but the tumor cyst wall still exists and is still regenerating^37,49,50^. The reason is that immunomodulation only inhibits the function of stem cell on the cyst wall, but does not better limit them. On the whole, on the premise of minimizing hypothalamic damage, reasonable management and control of cyst wall and stem cells may be the most active treatment. Although treatment strategies for craniopharyngiomas are controversial, researchers all understand the limitations of current treatment and are optimistic about the prospect of molecular biology research^5,37,51,52^. Therefore, based on the existence of stem cells, if the cyst wall is left after surgery, we should actively seek more effective treatment methods, including radiotherapy.

Strengths and limitations: the advantage of this study is to systematically reveal the molecular pathological characteristics of the cyst wall of ACP, and explained its clinical impact, which is of guiding value for clinical practice. The imperfection of this study lies in the small sample size due to the low incidence of craniopharyngioma^1,3^. We expect that a large sample, multicenter study can be performed in future studies, including the effect of radiotherapy on cyst wall stem cells.

## Conclusions

The cyst wall is an integral component of craniopharyngioma containing stem cell niches, and has the same histopathological and molecular characteristics as the solid component. The cyst wall is more representative of the biological characteristics of ACP. The residual cyst wall of ACP increases the risk of tumor recurrence. Considering the existence of stem cells, we should actively pay attention to the management of the cyst wall.

## Data Availability

The datasets generated and analysed during the current study are available in the Genome Sequence Archive (Genomics, Proteomics & Bioinformatics 2021) in National Genomics Data Center (Nucleic Acids Res 2022), China National Center for Bioinformation / Beijing Institute of Genomics, Chinese Academy of Sciences (GSA-Human: HRA002354) that are accessible at Shared URL: https://ngdc.cncb.ac.cn/gsa-human/browse/HRA002354.
